# Ontogenic mRNA expression of RNA modification writers, erasers, and readers in mouse liver

**DOI:** 10.1371/journal.pone.0227102

**Published:** 2019-12-31

**Authors:** Liming Chen, Pei Wang, Raman Bahal, José E. Manautou, Xiao-bo Zhong

**Affiliations:** 1 Department of Pharmaceutic Sciences, School of Pharmacy, University of Connecticut, Storrs, Connecticut, United States of America; 2 Department of Pharmacology, School of Basic Medical Sciences, Zhengzhou University, Zhengzhou, China; John Curtin School of Medical Research, AUSTRALIA

## Abstract

RNA modifications are recently emerged epigenetic modifications. These diverse RNA modifications have been shown to regulate multiple biological processes, including development. RNA modifications are dynamically controlled by the “writers, erasers, and readers”, where RNA modifying proteins are able to add, remove, and recognize specific chemical modification groups on RNAs. However, little is known about the ontogenic expression of these RNA modifying proteins in various organs, such as liver. In the present study, the hepatic mRNA expression of selected RNA modifying proteins involve in m^6^A, m^1^A, m^5^C, hm^5^C, m^7^G, and Ψ modifications was analyzed using the RNA-seq technique. Liver samples were collected from male C57BL/6 mice at several ages from prenatal through neonatal, infant, child to young adult. Results showed that most of the RNA modifying proteins were highly expressed in prenatal mouse liver with a dramatic drop at birth. After birth, most of the RNA modifying proteins showed a downregulation trend during liver maturation. Moreover, the RNA modifying proteins that belong to the same enzyme family were expressed at different abundances at the same ages in mouse liver. In conclusion, this study unveils that the mRNA expression of RNA modifying proteins follows specific ontogenic expression patterns in mice liver during maturation. These data indicated that the changes in expression of RNA modifying proteins might have a potential role to regulate gene expression in liver through alteration of RNA modification status.

## Introduction

Epigenetic regulation refers to the molecular events where gene expression is regulated without alterations in the DNA sequence [1]. Epigenetic regulation has been recognized as an extra level of the genetic codes with diverse mechanisms [2]. Previous studies have identified several epigenetic mechanisms, including chromatin remodeling, DNA methylation, histone tail modifications, and non-coding RNAs [3]. Regulation of these epigenetic codes, or called epigenomes, is critical for multiple biological processes, including cell division, reprogramming, and differentiation, which are vital for tissue and organ development [4, 5]. Furthermore, dysregulation or aberrant alterations of the above epigenetic mechanisms have been shown to be associated with developmental abnormalities, biological disorders, and diseases [6, 7]. The accumulating evidence of how important epigenetic modifications are to human health also calls for an increasing demand of epigenetic studies.

The diverse post-transcriptional modifications of RNAs, or called epitranscriptomics, have been recognized as another key player in epigenetic regulation mechanisms [8]. The pioneer studies of RNA chemical modifications were performed more than 50 years ago. Owing to the development of advanced biotechnological techniques, including next-generation sequencing and mass spectrometry techniques, the studies of RNA modification and epitranscriptomics have gained popularity again in recent years. Up to date, more than 100 RNA modifications have been identified in multiple RNA types from almost all known living organisms, including messenger RNAs (mRNAs), housekeeping non-coding RNAs, which include transfer RNAs (tRNAs) and ribosomal RNAs (rRNAs), and regulatory non-coding RNAs, which include microRNAs (miRNAs) and long non-coding RNAs (lncRNAs) [9]. These modifications have been shown to regulate processing or metabolism of RNAs, including RNA splicing, translocation, stability, and translation efficiency [9]. However, the studies of RNA modifications are still immature, and some recent researches are focused on mapping of RNA modifications, elucidating the biological roles of these modifications, and identifying what molecules are involved in these processes [10, 11].

Previously, the RNA modifications and epitranscriptomics were regarded as a relatively static status for a specific RNA structure. Whereas, recent studies have shown that the modifications of RNAs are a dynamic and reversible process [9]. Similar to other epigenetic mechanisms, the RNA modifications are also controlled by the “writers, erasers, and readers” proteins. Writers are proteins being capable of adding chemical groups to specific sites of RNA molecules, erasers are proteins to remove the modified chemical groups added by writers, and readers are a group of proteins with specialized domains, which can recognize and bind to the modified RNA sites. These proteins work together as a complex network in the regulation of dynamic RNA modifications. Furthermore, dysregulation and mutation of these currently known RNA modification proteins have been shown to be related to human diseases, including cardiovascular diseases, metabolic diseases, neurological disorders, and cancer [12, 13]. These findings illustrate the importance of studying the expression and function of RNA modification proteins.

Ontogenic development is a complicated process involving the buildup of genetic and epigenetic signatures [14]. This phenomenon is applicable to specific organs, including liver, which is the key organ in the metabolism of both endogenous and exogenous compounds. The development of the liver and its functions is critical to protect infants and children from exposure to environmental toxicants. The ontogenic expression patterns of several liver-specific genes during development have been reported [15, 16]. There is an increasing amount of evidence showing that epigenetic mechanisms are contributing to the regulation of ontogenic expression of genes, where the expression and function of epigenetic modifying proteins are key players [17–19]. However, whether RNA modifications, as relatively new epigenetic mechanisms, are also involved in the regulation of liver development and maturation is still largely unknown.

In the current study, the ontogenic mRNA expression of RNA modification proteins involved in several frequent RNA modifications, including N^6^-methyladenosine (m^6^A), N^1^-methyladenosine (m^1^A), 5-methylcytosine (m^5^C), 5-hydroxymethylcytosine (hm^5^C), N^7^-methylguanosine (m^7^G), and pseudouridine (Ψ), was studied in mouse liver at different ages during postnatal maturation using RNA sequencing.

## Materials and methods

### Animal experiments, total RNA extraction, cDNA library construction, and RNA-Seq

The procedures including animal experiments, RNA extraction, cDNA library construction, and RNA sequencing were described in previous publication [20].

### RNA-Seq data analysis

The RNA-Seq reads from the FASTQ files were mapped to the mouse reference genome (NCBI37/mm9) and the splice junctions were identified by TopHat 1.2. The output files in BAM (binary sequence alignment) format were analyzed by Cufflinks 1.0.3 to estimate the transcript abundance.

### Data visualization and presentation

The selected RNA modification enzyme genes were retrieved from Cufflinks output for further analysis. Significant gene expression was determined by the drop-in-deviance F test of the fitted FPKM data to a generalized linear model with a Poisson link function, a statistic designed to measure the significance of a gene’s measured FPKM value relative to a zero FPKM value. Data were presented as the mean ± S.D. Ontogenic expression patterns were presented using the GraphPad Prism 7 software program (GraphPad Software, Inc., La Jolla, CA).

## Results

### Ontogeny of mRNA expression of writers, erasers, and readers involved in *N*^6^-methyladenosine (m^6^A)

N^6^-methyladenosine involves the addition of a methyl group at the N-6 position of an adenosine base. High-throughput sequencing following m^6^A-specific antibody immunoprecipitation has revealed a high prevalence of this modification in multiple RNA types across different species. The m^6^A is believed to be the most abundant RNA modification in mRNAs, but it also exists in other RNA types, including miRNAs, lncRNAs, tRNAs, rRNAs, and other small RNAs [21–23]. The m^6^A modification has been shown to play a role in multiple biological processes, including development, metabolism, and circadian rhythm [24, 25], through regulation of RNA metabolism, transport, and translation [26–28].

**Fig 1:**
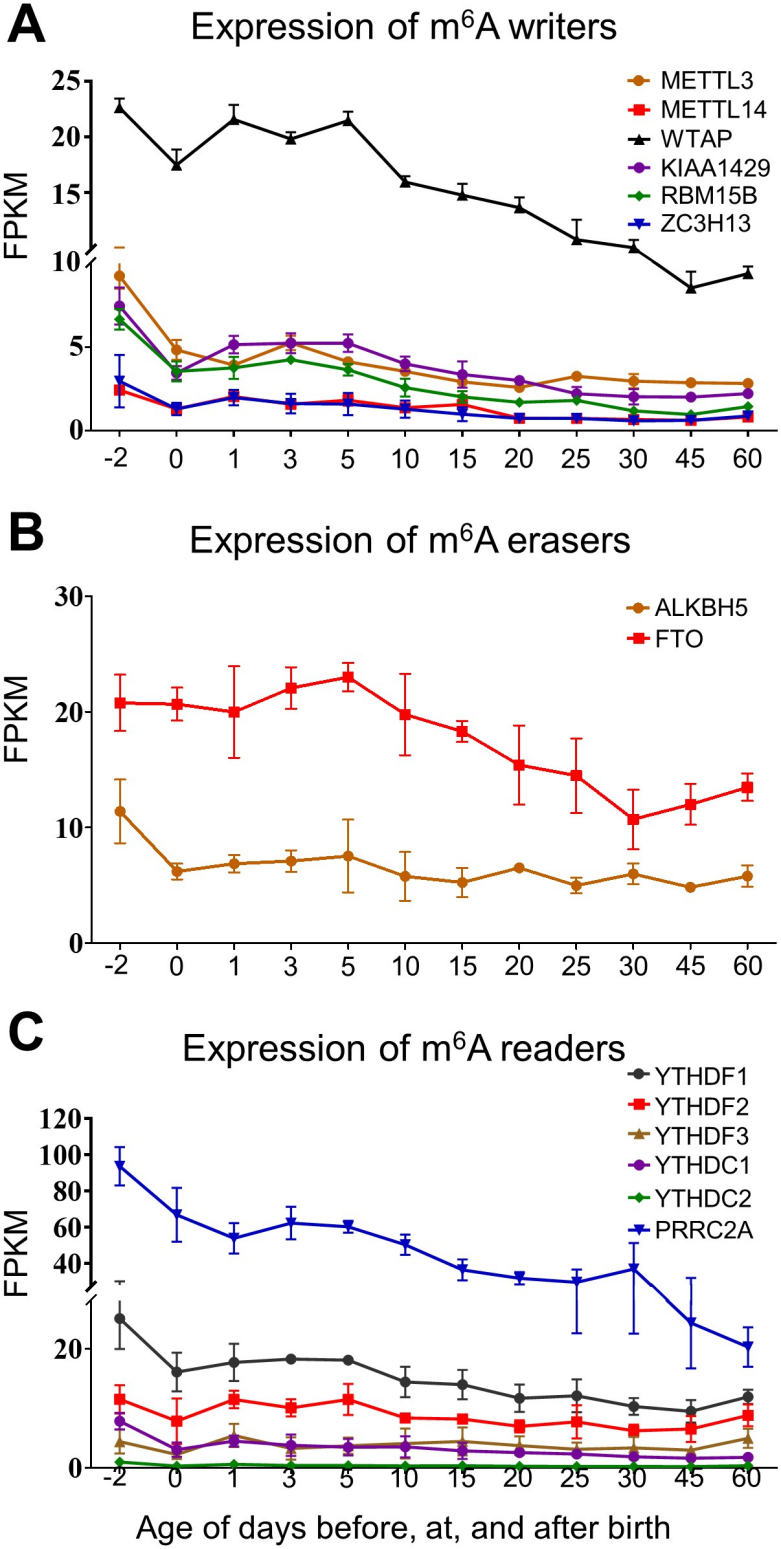
Hepatic ontogeny of mRNAs of genes involved in m^6^A modification. Livers were collected from male C57BL/6 mice at day -2 to 60. (A) mRNA expression of m^6^A writer genes. (B) mRNA expression of m^6^A eraser genes. (C) mRNA expression of m^6^A reader genes. Data are depicted as mean ± SD, n = 3. Y-axis represents mRNAs expressed as fragments per kilobase of exon per million reads mapped (FPKM).

The addition of m^6^A is accomplished by a methyltransferase complex (writers) consisting of several components, including methyltransferase Like 3 (METTL3), METTL14, Wilms tumor 1 associated protein (WTAP), Vir like m^6^A methyltransferase associated (VIRMA or KIAA1429), RNA binding motif protein 15 (RBM15), and zinc finger CCCH domain-containing protein 13 (ZC3H13). Each component in the complex has specific roles in the installment of m^6^A modification at a precise location [29–34]. As showed in Fig 1A, the expression of m^6^A writers followed specific trends. The expressions of METTL14 and ZC3H13 were the lowest among all tested components in the m^6^A methyltransferase complex. Compared to the prenatal expression, both METTL14 and ZC3H13 mRNA levels dropped ~50% at birth (Fig 1A). The postnatal expressions of METTL14 and ZC3H13 showed a constant drop until day 20 and 15, respectively (Fig 1A).

The expression of METTL3, KIAA1429, and RBM15B showed higher mRNA expression levels in mouse liver compared to METTL14 and ZC3H13. However, similar to METTL14 and ZC3H13, the expression of METTL3, KIAA1429, and RBM15B at birth decreased to only half of the prenatal levels. KIAA1429 showed a constant decrease during development after birth, whereas METTL3 and RBM15B were relatively stable between day 20 and 60 (Fig 1A). The expression of WTAP was highest among all tested components in m^6^A methyltransferase complex. The expression of WTAP dropped at birth, rebounding during day 0 to 5 after birth. After day 5, WTAP showed a constant sharp drop until adulthood.

The dynamic regulation of m^6^A is also controlled by another group of demethylases (erasers). So far, two enzymes have been identified as demethylases for the m^6^A modification, including fat mass obesity-associated protein (FTO) and AlkB Homolog 5 RNA Demethylase (ALKBH5) [27, 35–37]. Furthermore, dysregulation of these erasers has been shown to induce many types of disorders, indicating the important role of homeostatic m^6^A status in health. As shown in Fig 1B, the expression of ALKBH5 had a 45% decrease at birth, remaining stable after birth. The expression FTO had three phases, where the expression level increased slightly before day 5 postnatal, and decreased from day 5 to 30, followed by another increase until day 60 (Fig 1B).

Most m^6^A-mediated cellular functions depend on reader proteins, which can recognize and bind to m^6^A-modified RNAs. The YTH domain containing proteins are the best characterized m^6^A readers [38, 39]. Recruitment of reader proteins has different impacts on the target RNA molecules. Recruitment of YTHDF1 and YTHDF3 mainly regulates translation process. YTHDF2 mainly regulates RNA stability [40, 41]. Two additional members of the YTH protein family, the YTHDC1 and YTHDC2, were also detected in mice liver across different ages. According to previously reported, YTHDC1 was able to influence mRNA splicing process by affecting the function of pre-mRNA splicing factors [26]. In terms of YTHDC2, the protein was initially found to be important in spermatogenesis in male mice, through mechanisms including translation efficiency enhancement [42, 43]. A more recent study also showed that YTHDC2 had the potential to regulate mRNA stability through interaction with exoribonuclease XRN1, which indicated a more general role of YTHDC2 in gene regulation [44]. However, that are the roles of YTHDC2 in liver function is still largely unknown. The proline rich coiled-coil 2A (PRRC2A) is a recently discovered m^6^A reader protein, involved in glial development in mice [45]. The results in Fig 1C showed that the expression of PRRC2A was the highest among all the tested m^6^A readers in mouse liver. The expression of PRRC2A had a constant decrease during liver development. At day 60, the PRRC2A expression was only 20% compared to prenatal expression. YTHDF1 was the most abundant YTH domain containing reader, followed by YTHDF2, YTHDC1, YTHDF3, and YTHDC2. Regardless of the differences in expression levels, all these YTH domain containing readers showed a sharp decrease in expression at birth, ranging from 70% decrease (YTHDC2) to 32% decrease (YTHDF2) (Fig 1C).

### Ontogeny of mRNA expression of writers, erasers, and readers involved in *N*^1^-methyladenosine (m^1^A)

The N^1^-methyladenosine consists of addition of a methyl group at the N1 position of adenosine. The m^1^A modification was first identified in tRNAs and rRNAs decades ago [46]. Recently, transcriptome-wide mapping also revealed the existence of m^1^A modification in mRNAs [47, 48]. The m^1^A modification in tRNAs was important for tRNA folding, stability, and tRNA-protein interaction [49–52]. The m^1^A modification was also observed at several sites in rRNAs, which affect the tertiary structure of the ribosome and downstream gene translation [53, 54]. In contrast to non-coding RNAs, m^1^A modification has a relatively low abundance in mRNAs, with a potential function in regulating translation process [47].

**Fig 2:**
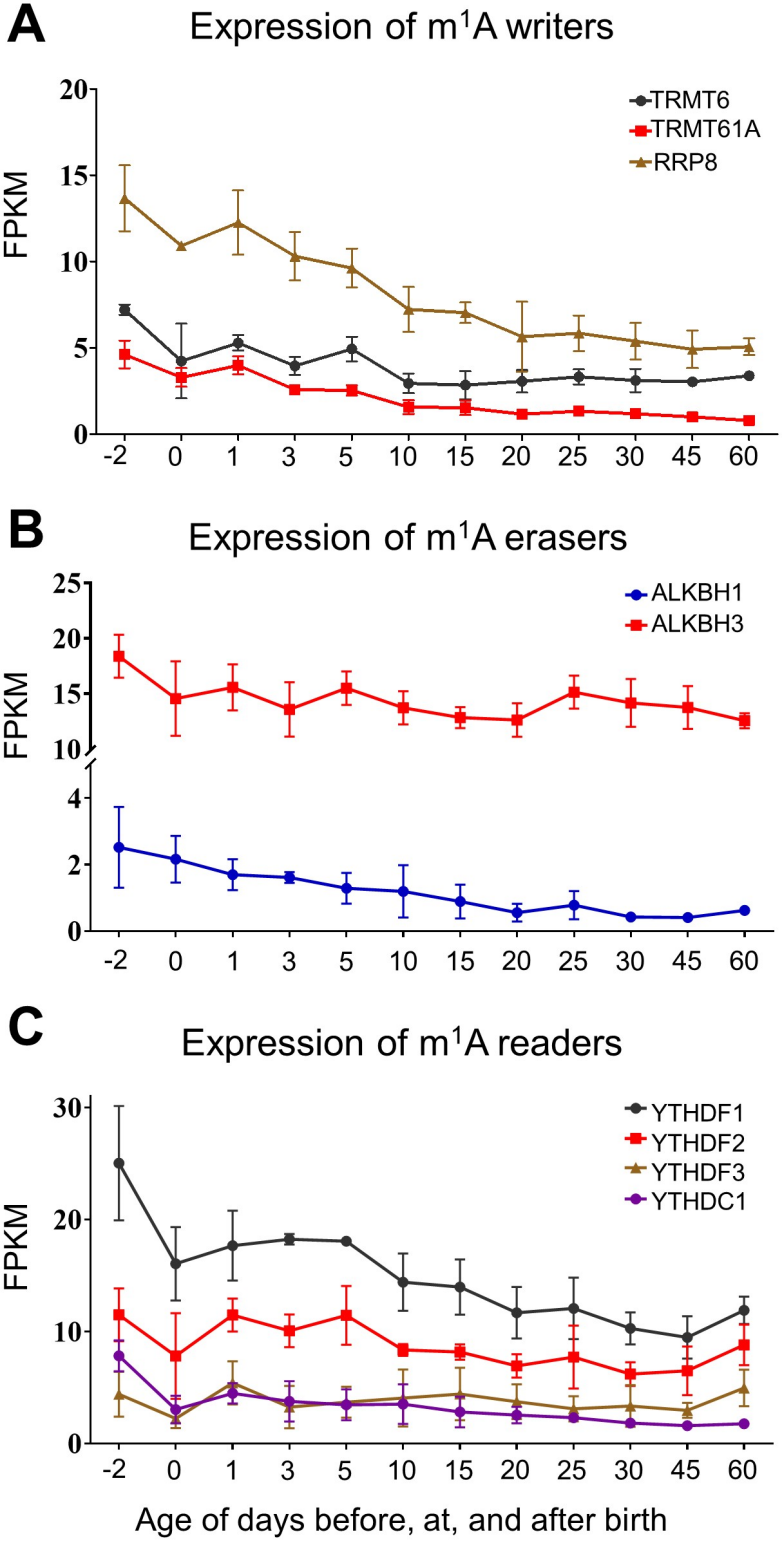
Hepatic ontogeny of mRNAs of genes involved in m^1^A modification. Livers were collected from male C57BL/6 mice day -2 to 60. (A) mRNA expression of m^1^A writer genes. (B) mRNA expression of m^1^A eraser genes. (C) mRNA expression of m^1^A reader genes. Data are depicted as mean ± SD, n = 3. Y-axis represents mRNAs expressed as fragments per kilobase of exon per million reads mapped (FPKM).

TRM6 and TRM61A are two identified m^1^A methyltransferases responsible for tRNA modification [55]. Nucleomethylin (NML or RRP8) was reported to catalyze m^1^A modification at multiple rRNA sites in both human and mouse cells [56]. These known tRNA/rRNA methyltransferases were also reported to be able to modify mRNAs, but specific mRNA m^1^A writers remain unknown. As shown in Fig 2A, the expression of all three m^1^A writers showed a decrease trend during development. The expression of ribosomal RAN-processing protein (RRP8) was the highest among all tested m^1^A writers and it decreased constantly in mouse liver with the highest level at the prenatal age. The expression of TRM6 decreased from prenatal day 2 to postnatal day 10 to about 40%, followed by a relatively stable expression until adulthood. The expression of TRM61A dropped constantly, but the dropping rate was reduced after postnatal day 10.

Two proteins in the AlkB family have been identified to be m^1^A demethylases, ALKBH3 and ALKBH1 [48, 57]. Overall, the expression of ALKBH1 decreased during mouse liver development, with some elevations at days 1, 5, and 25 after birth (Fig 2B). The expression of ALKBH3 was much lower compared to ALKBH1 with a constant decrease at all tested ages (Fig 2B).

The YTH-domain containing proteins YTHDF1, YTHDF2, YTHDF3, and YTHDC1, but not YTHDC2, are also reported to be m^1^A reader proteins, which have a role in translation regulation [58]. The expression patterns of m^1^A readers (Fig 2C) were the same to that of m^6^A readers, which were presented above in Fig 1C.

### Ontogeny of mRNA expression of writers, erasers, and readers involved in 5-methylcytosine (m^5^C)

The 5-methylcytosine (m^5^C) modification was first identified in DNA molecules, but also found later in RNAs [59, 60]. Utilizing transcriptome wide mapping technique, the m^5^C modification has been identified to be widely distributed in multiple types of RNAs, both coding and non-coding [61, 62]. Known functions of m^5^C modification includes regulation of translation, RNA metabolism, and RNA trafficking [63–66].

**Fig 3:**
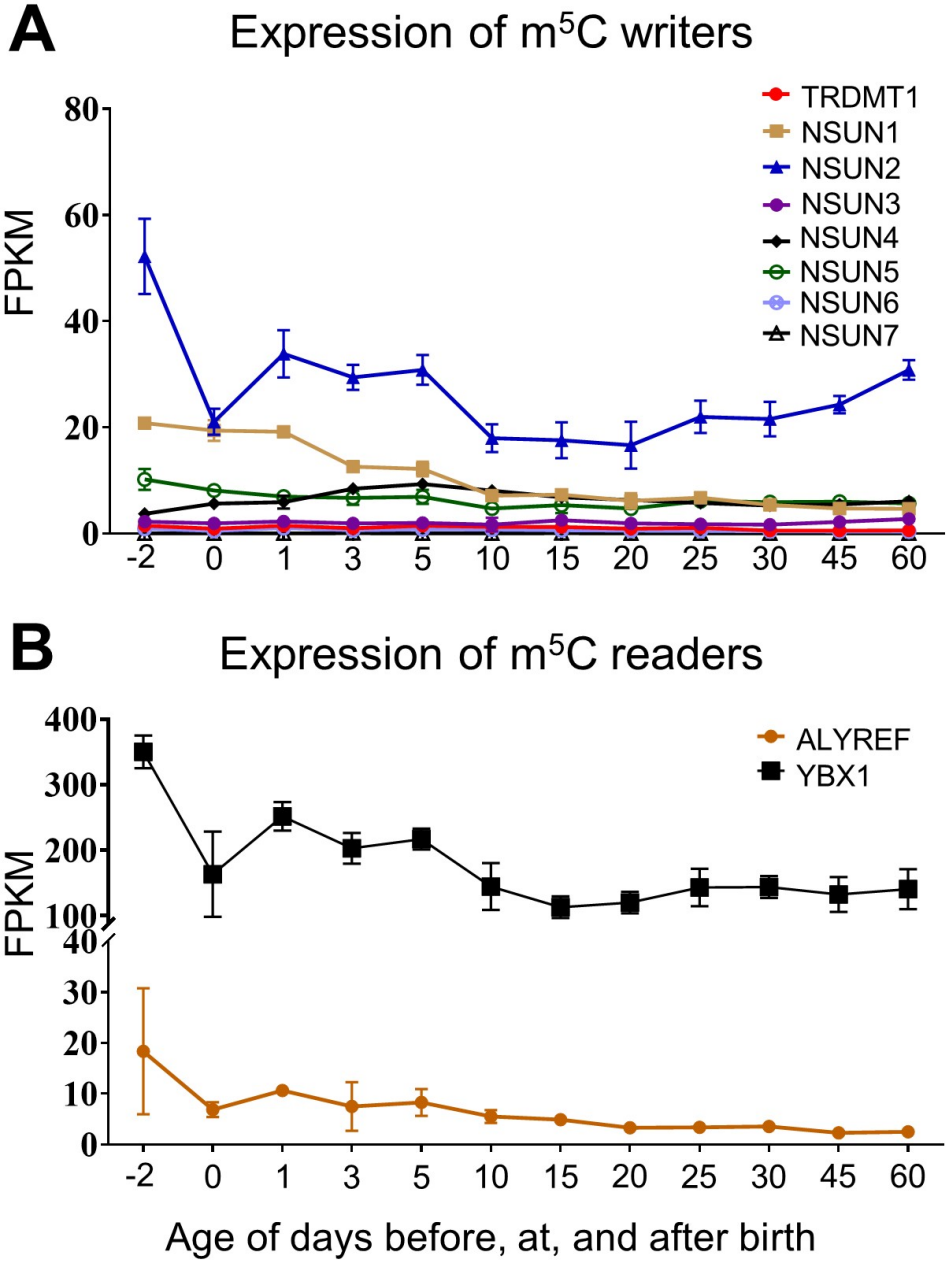
Hepatic ontogeny of mRNAs of genes involved in m^5^C modification. Livers were collected from male C57BL/6 mice day -2 to 60. (A) mRNA expression of m^5^C writer genes. (B) mRNA expression of m^5^C reader genes. Data are depicted as mean ± SD, n = 3. Y-axis represents mRNAs expressed as fragments per kilobase of exon per million reads mapped (FPKM).

The RNA m^5^C methyltransferases (MTases), which contain S-adenosyl-L-methionine (SAM)-dependent MTase domains, are the major writers of m^5^C modification. Several members of the NOP2/Sun RNA methyltransferase family (NSUN) have been identified to methylate different RNA molecules [66, 67]. Among the tested NSUNs, NSUN2 had the highest expression (Fig 3A). The expression of NSUN2 dropped 60% at birth, but rebounded during postnatal day 0 to 10. At day 10, NSUN2 was expressed at similar levels compared to day 0 and started to increase in liver until adulthood. NSUN2 was reported to mediate mammalian mitochondrial tRNAs in several different positions and involve in processes including cell differentiation and motility [68–70]. The expression of NSUN1 (or NOP2) was the second highly expressed NSUNs following NSUN2, which showed a constant decrease from prenatal to adulthood. Unlike NSUN2, NSUN1 was reported to be a methyltransferases for rRNAs and regulate processes including cell proliferation or drug response [71, 72]. NSUN4 and NSUN5 were expressed in a relatively stable manner across all tested ages. Both NSUN4 and NSUN5 were able to methylate rRNAs. NSUN4 was able to methylate 12S rRNA and played a key role in ribosome biogenesis while NSUN5 was able to methylate 25S rRNA and regulate lifespan and differential stress response in yeast [73, 74]. Three members of the NSUNs, the NSUN3, NSUN6, and NSUN7 were barely expressed in mice liver across all tested ages (Fig 3A). Other than NSUNs, the tRNA aspartic acid MTase 1 (TRDMT1 or DNMT2), which was previously believed to be a DNA methyltransferase, was also reported to mediate the formation of m^5^C modification in tRNAs [75, 76]. However, the detected TRDMT1 expression was very low in mouse liver at all ages.

The study of demethylase of m^5^C is still very limited. There are currently no identified proteins, which can remove the methyl group at m^5^C modified sites. But it has been reported that m^5^C modification can be converted to other types of modifications, for example hm^5^C [77]. This indicates that the erase or elimination of m^5^C might be accomplished by further modification of the methyl group instead of removing it.

The Aly/ REF export factor (ALYREF) is the first identified m^5^C reader protein, which regulates the exportation of m^5^C modified mRNAs [65]. As shown in Fig 3B, compared to prenatal expression, the expression of ALYTEF dropped sharply to 37% at birth. Its expression kept a decreasing trend until adulthood, at which age the expression level was just 14% the prenatal level. The Y-box binding protein 1 (YBX1) is another recently identified m^5^C reader protein, which was reported to promote the pathogenesis of human urothelial carcinoma of the bladder in an m^5^C-modification-dependent mechanism, where the binding of YBX1 maintained the stability of target mRNAs [78]. From Fig 3B, the overall expression level of YBX1 was much higher than ALYREF. But similarly, a 53% drop in expression level at birth was also observed in YBX1, followed by a rebound between postnatal day1 to day 10. After postnatal day 10, the expression showed a relative stable expression.

### Ontogeny of mRNA expression of writers and readers involved in 5-hydroxymethylcytosine (hm^5^C)

Similar to m^5^C, the hm^5^C was first identified in DNAs, which was later identified in mammalian RNAs [79]. However, recent studies on hm^5^C are still focused on its role in DNA modifications with very limited knowledge on RNA modifications. Studies have shown that the hm^5^C in the coding sequences of mRNAs increases translation efficiency through unknown mechanisms [80].

**Fig 4:**
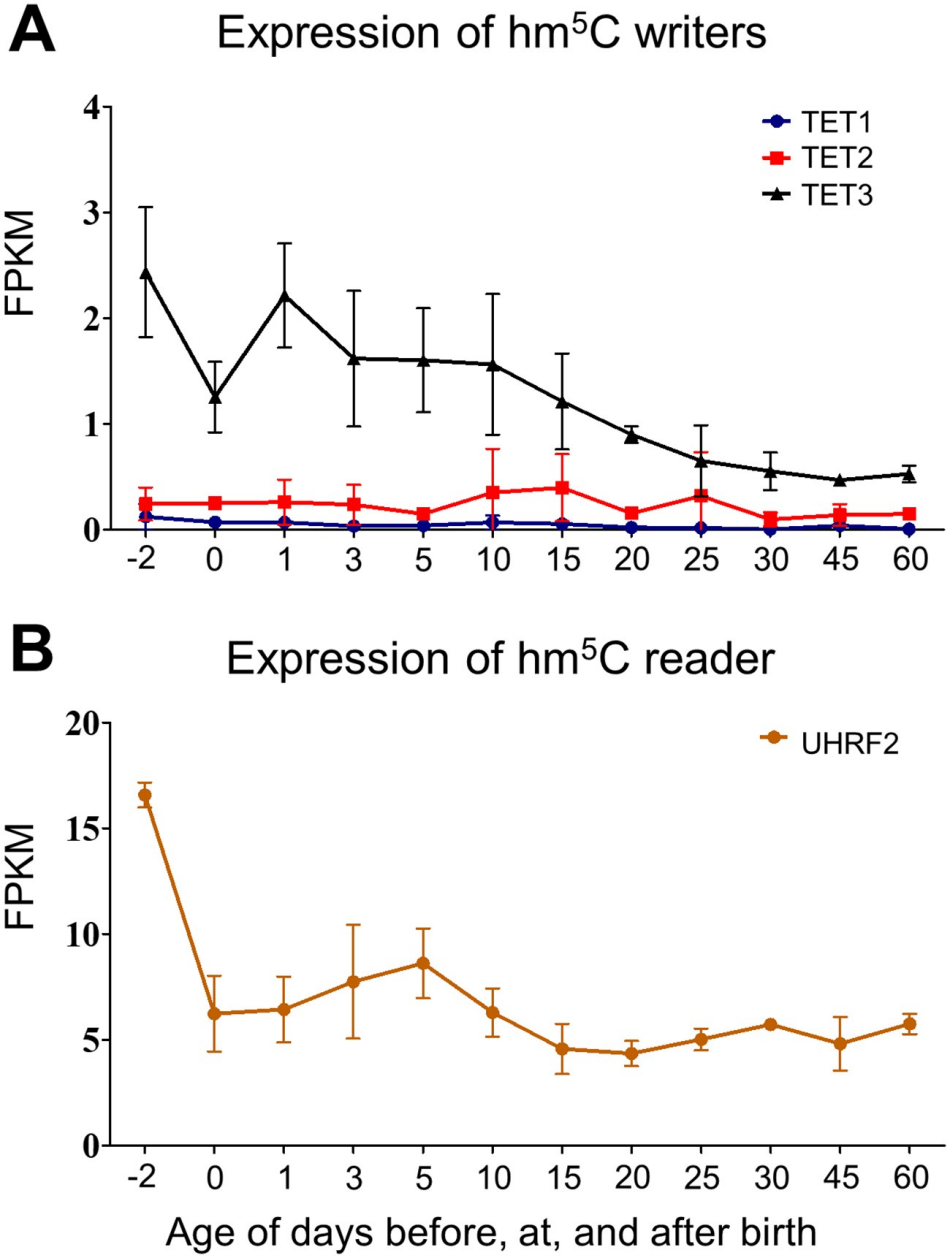
Hepatic ontogeny of mRNAs of genes involved in hm^5^C modification. Livers were collected from male C57BL/6 mice day -2 to 60. (A) mRNA expression of hm^5^C writer genes. (B) mRNA expression of hm^5^C reader genes. Data are depicted as mean ± SD, n = 3. Y-axis represents mRNAs expressed as fragments per kilobase of exon per million reads mapped (FPKM).

The ten-eleven translocation (TET) family proteins, including TET1, TET2, and TET3, were reported to mediate the oxidation of m^5^C to hm^5^C in both DNAs and RNAs [79]. As shown in Fig 4A, the expression of TET3 was the highest in mouse liver followed by TET2 and TET1. The expression of TET3 dropped to 50% at birth and rebounded to 90% at postnatal day 1. This was followed by a constant decrease during liver development. The expression of TET2 increased between postnatal days 5 to 30, where the peak at postnatal day 15 was 1.6-fold higher than the expression at prenatal day 2. The expression of TET1 dropped 43% at birth and remained low during liver development (Fig 4A). However, in a previous study, TET-null mouse embryonic stem cells also exhibited significantly high levels of hm^5^C in RNAs, which indicates the existence of hm^5^C writers other than TETs [79]. Currently, there are no identified hm^5^C erasers in RNAs. Future studies are still needed to cover this knowledge gap.

The ubiquitin-like with PHD and ring finger domains 2 (UHRF2) proteins might have the potential to bind to hm^5^C modified RNAs. The UHRF2 was reported to bind to hm^5^C modified DNAs [81, 82]. Fig 4B shows that the expression of UHRF2 dropped sharply at birth, with a 60% decrease comparing to prenatal day 2. Its expression increased slightly during postnatal days 0 to 5, followed by a constant drop until adulthood.

### Ontogeny of mRNA expression of writers involved in *N*^7^-methylguanosine (m^7^G)

N^7^-methylguanosine (m^7^G) is another most prevalent modifications existing in multiple types of RNAs, including tRNA, rRNA, and mRNA [83–86]. In tRNAs, catalyzed by the methyltransferase like 1 (METTL1)/WDR4 complex in human and mouse, m^7^G was reported to regulate the stability and decay pathways of tRNAs, which further involved in the regulation of physiological processes including cellular development and human disease [87–89]. The m^7^G is also conserved in rRNAs in multiples species. Catalyzed by the WBSCR22/TRMT112 complex, where the TRMT112 protein acts as a co-activator of the methyltransferases WBSCR22, m^7^G modification was reported to regulated the processing, export, and maturation of several pre-rRNAs and rRNAs [90, 91]. In mRNAs, m^7^G cap modification was first identified, which is mediated by the RNA (guanine-7-) methyltransferase (RNMT)/RAM methyltransferase complex, and regulate the export, stability, splicing, transcription, translation, and decay of mRNA [92–95]. Utilizing more advanced sequencing techniques, two more recent studies mapped the distribution of m^7^G in a transcriptome wide manner and found the existence of m^7^G in internal mRNAs and long non-coding RNAs, with a potential role in translational regulation [85, 86].

**Fig 5:**
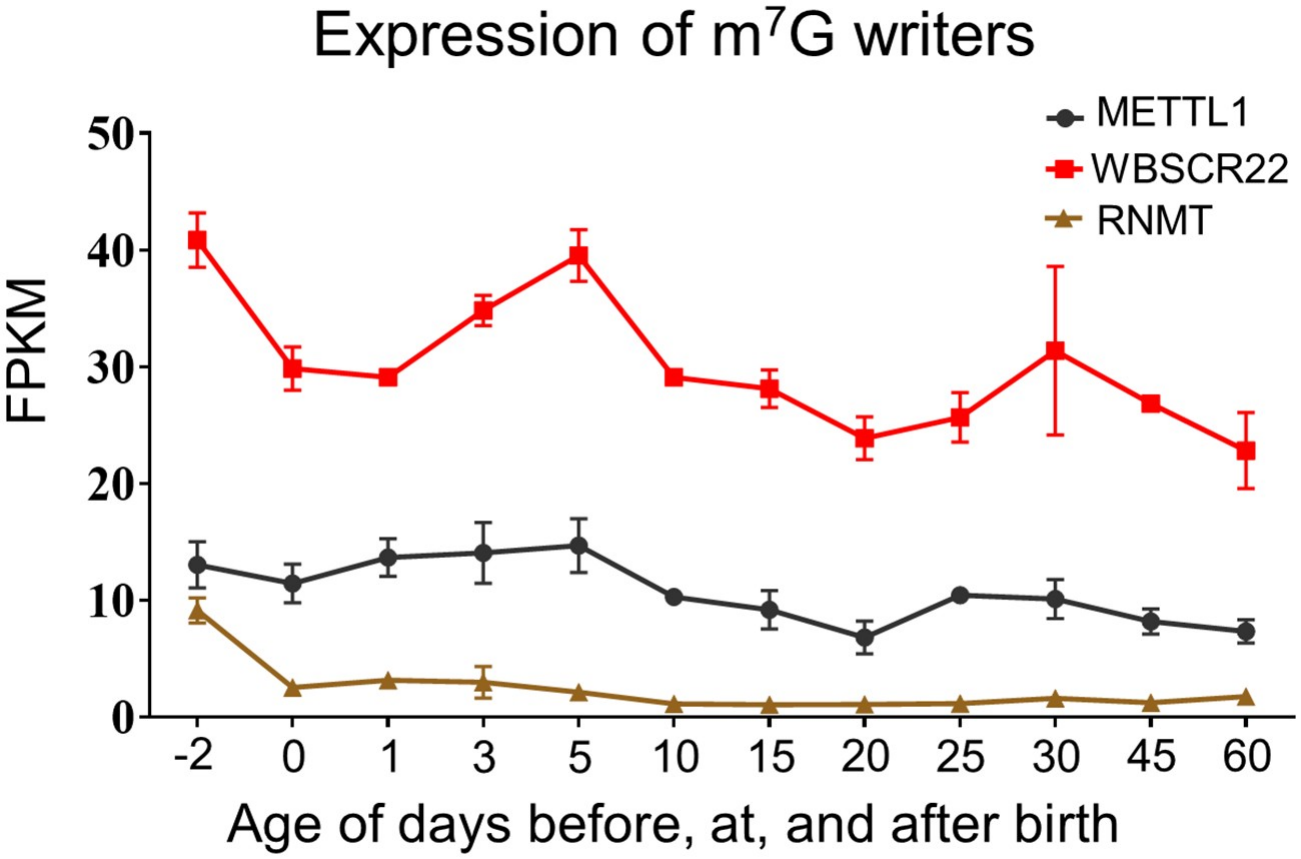
Hepatic ontogeny of mRNAs of genes involved in m^7^G modification. Livers were collected from male C57BL/6 mice day -2 to 60. mRNA expression of m^7^G writer genes. Data are depicted as mean ± SD, n = 3. Y-axis represents mRNAs expressed as fragments per kilobase of exon per million reads mapped (FPKM).

There are three identified writer enzymes of m^7^G modification. METTL1 was reported to catalyze the formation of m^7^G on both tRNAs and mRNAs. As showed in Fig 5, the expression of METTL1 was relatively stable during the development of mice across all tested ages. WBSCR22 is another reported m^7^G methyltransferase catalyzing the formation of m^7^G on pre-rRNAs and rRNAs. The expression of WBSCR22 dropped at birth and rebounded to its prenatal level at day 5, followed by another drop (Fig 5). RNMT is the enzyme mediating cap m^7^G modifications. The expression of RNMT was found highest in prenatal mice liver. At birth, the level of RNMT dropped and stayed low till adulthood.

The research on identifying specific reader and erasers of m^7^G are still limited and future studies are needed to address these knowledge gaps.

### Ontogeny of mRNA expression of writers involved in pseudouridine (Ψ)

Pseudouridine (Ψ), a C-C glycosidic isomer of uridine (U), is derived from the incorporation of C5 into the glycosidic bond [96]. Recent sequencing results have revealed the wide distribution of the Ψ modification in different species of RNAs [97, 98]. However, Ψ still predominantly exists in non-coding RNAs, tRNAs, and rRNAs, with relatively lower expression in mRNAs [99]. In tRNAs, the Ψ modification has been shown to regulate stability of tRNAs [100]. In rRNAs, the Ψ modification has been shown to regulate the ribosome assemble process, which is important to protein synthesis initiation [101]. The function of the Ψ modification in mRNAs is still largely unknown. The Ψ modification was found in multiple regions of mRNAs with no specification preference. Previous studies showed that the existence of the Ψ modification increases the stability of mRNAs against heat shock stress and is associated with higher translation efficiency [102, 103].

**Fig 6:**
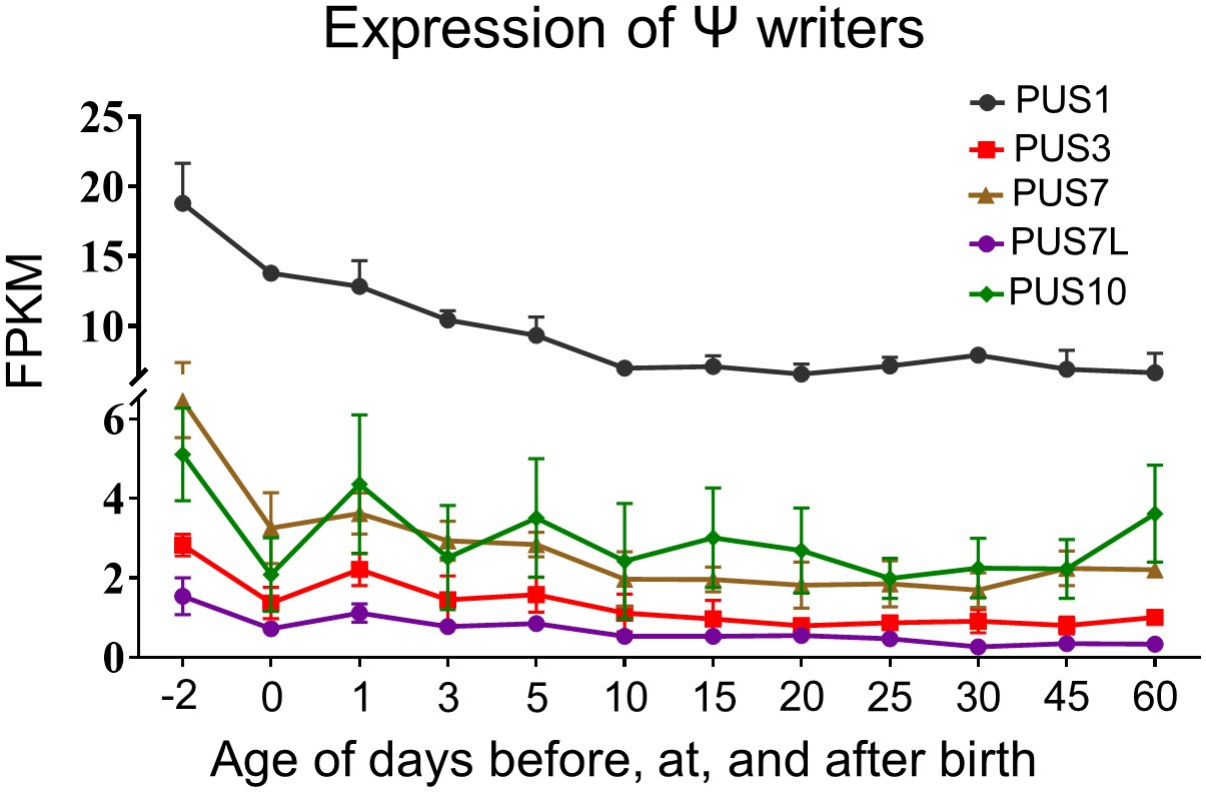
Hepatic ontogeny of mRNAs of genes involved in Ψ modification. Livers were collected from male C57BL/6 mice day -2 to 60. mRNA expression of Ψ writer genes. Data are depicted as mean ± SD, n = 3. Y-axis represents mRNAs expressed as fragments per kilobase of exon per million reads mapped (FPKM).

In RNAs, uridine is transformed into pseudouridine by a class of enzymes known as pseudouridine synthases (PUSes). Different PUSes have shown preferences for different residues in tRNAs or rRNAs. Several PUSes were identified in mouse liver by RNA-sequencing, as shown in Fig 6. The expression of PUS1 was the highest among all tested PUSes with a constant decreasing trend from prenatal day 2 to 10, followed by a relatively stable expression. At postnatal day 10, the expression level of PUS1 was only 47% compared to its peak level at prenatal day 2. The remaining PUSes, PUS3, PUS7, PUS7L, and PUS10 all showed a ~50% decrease at birth, in comparison to prenatal expression (Fig 6).

The erasers and readers of the Ψ modification are still largely unknown. Future studies are needed to fill in these knowledge gaps.

## Discussion

Even though RNA modifications have been known for decades, the biological significance of these modifications has only been recognized in recent years, owning to the development of genome-wide and high resolution sequencing techniques. Recent studies have identified more than 150 types of RNA modifications covering almost all RNA types in the genomes of different species [9]. Although the knowledge about the biological functions of these modifications is still limited, it has been shown that RNA modifications are able to regulate RNAs in multiple ways, including metabolism, transport, and translation.

Development involves dynamic regulation of gene expression in cells, where RNAs play a non-negligible role in this process. Chemical modifications of RNAs have emerged as promising mechanisms in modulation of development process. Previous studies have shown the importance of RNA modifying proteins in development. Deletion of *Mettl3*, the m^6^A writer gene, has been shown to be embryonically lethal in mice with dysregulation of pluripotency in embryonic stem cells [104]. Knockout of some m^6^A modifiers also affected neuronal development and reproductive function (fertility, spermatogenesis, and oogenesis) [105]. Aside from the m^6^A modification, some other types of RNA modifications are also involved in development regulation. Knockout of m^5^C modification writer *Nsun2* or *Dnmt2* has been reported to affect stem cell differentiation and endochondral ossification, respectively [106, 107]. All these findings underscore the importance of RNA modifications and RNA modifying proteins in the development process.

In the present study, the mRNA expression of genes involved in several of the most common RNA modifications, including m^6^A, m^1^A, m^5^C, hm^5^C, m^7^G, and Ψ, were studied in mouse liver at different ages during development. Most of these RNA modifying proteins showed ontogenic changes in mRNA levels along with liver development, where the majority of them showed dramatic drops at birth followed by downregulations during postnatal development. These data indicate that RNA modification status might also have such ontogenetic changes, which is regulated by the alterations in RNA modifying proteins.

The data also showed that most RNA modifying enzymes are high expressed in prenatal compared to postnatal mouse liver, at which stage the cells are more stem-like. The studies mentioned above have suggested the important role of RNA modifications in stem cell functions. So it may be interesting to study how the RNA modifying proteins are expressed in the early stages of prenatal development. However, in the current research, only prenatal day two liver samples were collected and future experiments are needed to analyze the prenatal expression of RNA modifying proteins.

Many other liver-specific genes have been reported to show specific ontogenic expression patterns during development, including phase I and phase II drug metabolizing enzymes [16, 108]. However, the factors contributing to this phenomenon are still largely unknown. The currently discovered RNA modifications might involve in this ontogenic regulation of gene expression. Moreover, discrepancies have been reported between mRNA and protein expression for multiple genes. This is very common for cytochrome P450 genes [109, 110]. As post-translational modification mechanisms, RNA modifications might be also responsible for this phenomenon. However, the study of RNA modifying proteins is not sufficient to confirm the involvement of RNA modifications in these biological events. The expression patterns of RNA modifying proteins reported here is not sufficient to explain all genes with different expression patterns. Direct detection of RNA modification types and sites on the RNA transcripts of the genes is still needed to study how RNA modifications involved in the regulation of a specific gene.

In summary, the present study provides new knowledge about the ontogenic mRNA expression of multiple RNA modifying proteins during development of mouse liver. Such knowledge can serve as foundation for future studies on the impact of RNA modifications in gene regulation during liver development. Understanding the biological significance of RNA modifications in liver development or functions will also benefit the study of drug metabolism or liver diseases in the future.
